# Cost utility of fractional exhaled nitric oxide monitoring for the management of children asthma

**DOI:** 10.1186/s12962-021-00287-3

**Published:** 2021-06-03

**Authors:** Jefferson Antonio Buendía, Ranniery Acuña-Cordero, Carlos E. Rodriguez-Martinez

**Affiliations:** 1 Department of Pharmacology and Toxicology, School of Medicine, Research Group in Pharmacology and Toxicology (INFARTO), Facultad de Medicina, Universidad de Antioquia, Carrera 51D #62-29, Medellín, Colombia; 2 Departamento de Neumología Pediátrica, Hospital Militar Central, Departamento de Pediatría, Facultad de Medicina, Universidad Militar Nueva Granada, Bogotá, Colombia; 3 Department of Pediatrics, School of Medicine, Universidad Nacional de Colombia, Bogota, Colombia; 4 Department of Pediatric Pulmonology and Pediatric Critical Care Medicine, School of Medicine, Universidad El Bosque, Bogota, Colombia

**Keywords:** Health economics, Public health, Healthcare

## Abstract

**Introduction:**

Fractional exhaled nitric oxide is a simple, non-invasive measurement of airway inflammation with minimal discomfort to the patient and with results available within a few minutes. This study aimed to evaluate the cost-effectiveness of asthma management using fractional exhaled nitric oxide monitoring in patients between 4 and 18 years of age.

**Methods:**

A Markov model was used to estimate the cost-utility of asthma management using fractional exhaled nitric oxide monitoring versus asthma management without using fractional exhaled nitric oxide monitoring (standard therapy) in patients between 4 and 18 years of age. Cost data were obtained from a retrospective study on asthma from a tertiary center, in Medellin, Colombia, while probabilities of the Markov model and utilities were obtained from the systematic review of published randomized clinical trials. The analysis was carried out from a societal perspective.

**Results:**

The model showed that fractional exhaled nitric oxide monitoring was associated with a lower total cost than standard therapy (US $1333 vs. US $1452 average cost per patient) and higher QALYs (0.93 vs. 0.92 average per patient). The probability that fractional exhaled nitric oxide monitoring provides a more cost-effective use of resources compared with standard therapy exceeds 99% for all willingness-to-pay thresholds.

**Conclusion:**

Asthma management using fractional exhaled nitric oxide monitoring was cost-effective for treating patients between 4 and 18 years of age with mild to moderate allergic asthma. Our study suggests evidence that could be used by decision-makers to improve clinical practice guidelines, but this should be replicated in different clinical settings.

## Introduction

The periodic assessment and early management of airway inflammation in patients with asthma are the principal strategies to prevent hospitalizations as recommended by international and local clinical guidelines [1]. The frequent measure of airway inflammation during monitoring plays an important role in anticipating exacerbations and optimizing the use of biological and corticosteroid drugs [2, 3].

Fractional exhaled nitric oxide (FeNO) may be a surrogate marker for type 2 airway inflammation [2]. FeNO is a simple, non-invasive measurement of airway inflammation with minimal discomfort to the patient and with results available within a few minutes. FeNO correlates with airway eosinophilia in biopsy and bronchoalveolar lavage fluid [3]. In fact, a meta-analysis of eight clinical trials in children found that FeNO-guided treatment reduced asthma exacerbations [4]. However, the routine use of FeNO in asthma and in children has not been uniformly adopted by all countries, especially by developing countries.

For policymakers, the main barrier to adopting new technologies is always doubt about their efficiency in scenarios with scarce health resources. Different economic evaluations of the use of FeNO during asthma management in developed countries have demonstrated that FeNO monitoring to guide asthma management was cost-effective in Spain, Germany, the UK, and the US [5–8]. In this paper, we aimed to evaluate the cost-effectiveness of asthma management using fractional exhaled nitric oxide monitoring in patients between 4 and 18 years of age.

## Material and methods

### Economic model

**Fig. 1:**
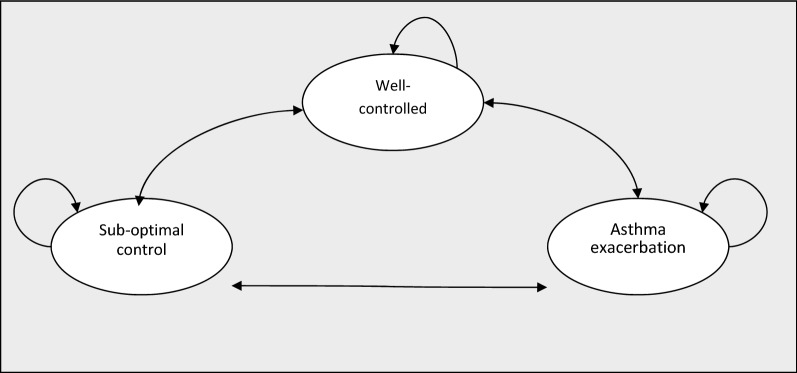
Markov model

A Markov simulation model with three mutually exclusive non-absorbent states was used to compare the estimated cost and outcomes associated with asthma management using fractional exhaled nitric oxide monitoring (FeNO) versus asthma management without using fractional exhaled nitric oxide monitoring (standard therapy) (Fig. 1). According to the natural history, three health states were defined: “no symptoms or asthma controlled”, “suboptimal control without exacerbation”, and “asthma exacerbation” with a cycle length of 1 week. All patients entering the model were children with no symptoms, diagnosed with mild to moderate allergic asthma, and receiving inhaled corticosteroids as maintenance therapy. The analysis was carried out from a societal perspective (it included direct and indirect costs). The analytic horizon was 12 months. Discount rate was not applied to estimate the present value of future cost and QALYs given the shorter time horizon. The study protocol was reviewed and approved by the Institutional Review Board of the University of Antioquia (No. 18/2015).

### Probabilities of Markov model and utilities

Probabilities of Markov model and utilities were obtained from a systematic review of randomized clinical trials, systematic reviews and previous economic evaluations. Searches of computerized databases (MEDLINE, EMBASE, CENTRAL, and LILACS) and references cited in published literature identified potentially applicable studies. The structured literature searches in these databases were made using the following criteria: (asthma OR wheeze) and (“Nitric Oxide”[tiab] or FeNO or eNO “exhaled NO”[tiab]). The searches yielded 493 citations and a total of 54 studies were examined in full. Studies comparing the adjustment of asthma medications based on FeNO to management based on clinical symptoms or current asthma guidelines or both were included. We excluded studies with the following comorbidities/characteristics: eosinophilic bronchitis, asthma related to underlying lung diseases such as bronchiectasis and chronic obstructive pulmonary disease or diagnostic categories such as 'cough variant asthma' and 'wheezy bronchitis'.

Finally, after applying these criteria, data from the utilities and transition probabilities were extracted from 1 previous study that evaluates the cost-utility of inhaled steroids in pediatric asthma within national guidelines for pediatric asthma [9], 8 randomized clinical trials [10–18], and 1 systematic review [4] (Table 1).

**Table 1:** Model inputs

Model input	Base case value	Distribution	References
Transition probabilities			
W to S	0.097	β(SD: 0.029)	[9]
W to A	0.004	β(SD: 0.002)	
S to W	0.817	β(SD: 0.038)	
S to A	0.007	β(SD: 0.003)	
A to W	0.271	β(SD: 0.044)	
A to S	0.052	β(SD: 0.046)	
Utility			
Well-controlled	0.99	β(SD: 0.016)	[9]
Sub-optimal control	0.70	β(SD: 0.072)	
Asthma exacerbation	0.31	β(SD: 0.070)	
FeNO-SC effectiveness			
Relative risk of reduction of exacerbations	0.76	LogN(SD: 0.274)	[10–18]

Well-controlled: W

Sub-optimal control: S

Asthma exacerbation: A

**Fig. 2:**
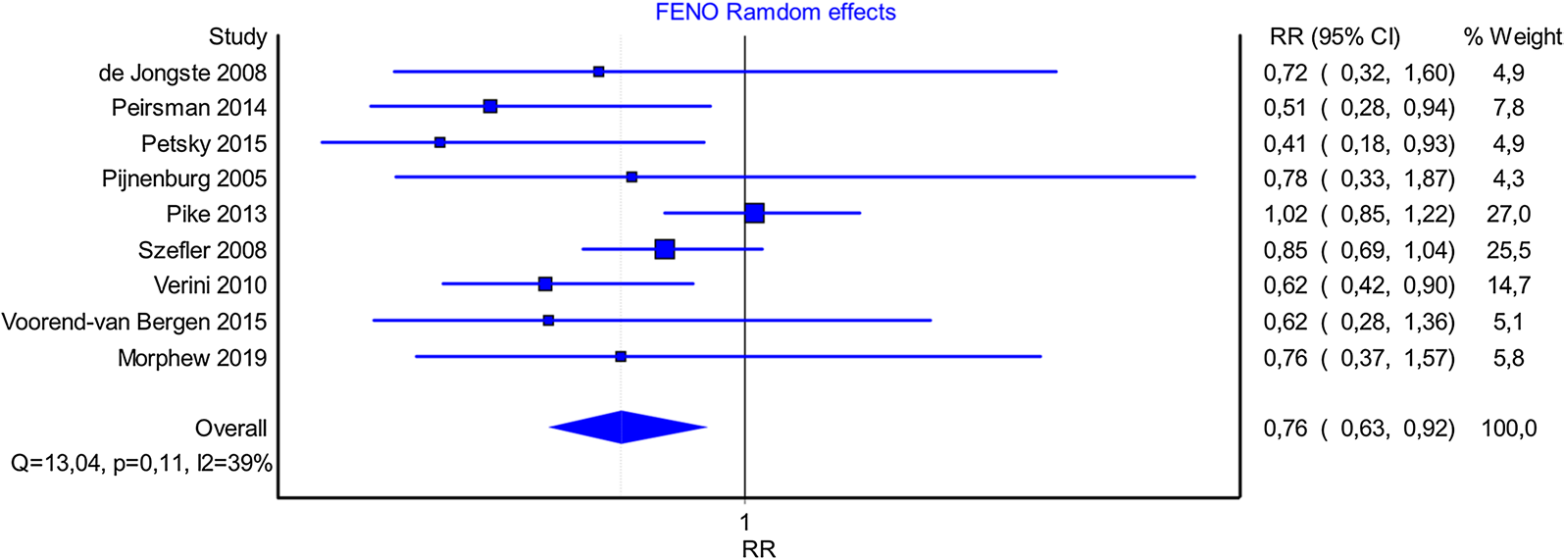
Forest plot of RCTs included

To estimate the relative risk of reducing the risk of exacerbation of FENO compared to standard therapy, a random-effect meta-analysis of the 8 eligible studies was performed to summarize their results. The meta-analysis was based on the DerSimonian and Laird method. The analysis found a reduced risk of exacerbation (RR 0.76 CI 0.63–092, I^2^ 39%), between the exacerbation rates observed by FeNO (Fig. 2). This relative risk was applied to the probability of hospitalization associated with standard therapy to derive the probability of hospitalization in the FENO group.

### Cost analysis

To estimate the cost of each health state defined in the model, we extracted all costs of infants under 18 years of age in Colombia, due to asthma according to the national clinical guideline of asthma in children from study previously published [19]. In brief, all costs and use of resources were collected directly from medical invoices and electronic medical records. The direct costs considered in the analysis include medical consultation at the emergency room, specialist referrals, chest physiotherapy, diagnosis support (laboratory, electrocardiogram, X-ray, etc.), medication (oxygen, nebulization, antibiotics, corticosteroids, bronchodilators, etc.), medical devices, accommodation services at intensive care units, and accommodation services in general medical wards (Table 2). Our country has been characterized by having very low price variation in the last 10 years, especially in health services between different clinics and over time [20]. Moreover, the proportion of each of the costs remains relatively constant, with few variations in their composition in the last 10 years [20]. We use US dollars (currency rate: US$ 1.00 = COP$ 3,000) [21] to express all costs in the study. For the valuation of the indirect costs associated with parents’ loss of productivity, the human capital method was used, assuming everyone receives an income of at least legal minimum wage for formal or informal work. The cost-opportunity of the productivity loss at the workplace and the caregiver was assessed based on the minimum wage without including transportation assistance for 2019 (US$ 229.81 per month). The government-approved legal minimum wage was taken as a reference instead of an average or median wage thereof as over 75% of the Colombian population earns minimum wage [22]. Since all the patients with asthma included in this study were children, we assumed that at least one family member accompanied the patient permanently during hospitalization, since pediatric hospitals in the country usually only allow one companion per patient in the hospital. The cost associated with transportation and food (not including an overnight stay) was assumed to correspond to 50% of minimum wage per day.

**Table 2:** Cost used in base case and sensitivity analyses

Model input	Base case value	SA range for one-way sensitivity analyses	Distribution
Intervention cost			
FeNO per patient day	2.20	1.20–4.20	γ(SD:1.08)
Hospitalization cost			
Daily cost in pediatric ward	95.05	80.23–102.01	γ(SD:8.53)
Hospital length of stay (days)	5.50	4.00–8.00	γ(SD:1.04)
PICU related cost			
Daily cost in PICU	406.52	430.26–350.43	γ(SD:18.89)
PICU lenght of stay (days)	10.9	7.75–15.05	γ(SD:3.26)
Emergency visit prior hospitalization cost			
Daily cost of emergency ward	64.3	51.19–71.46	γ(SD:19.27)
Direct medical cost per patient-day			
Specialist referrals	10.67	10.31–11.01	γ(SD:1.72)
Chest physiotherapy	5.15	4.90–5.39	γ(SD:1.23)
Chest radiography	2.84	2.70–2.98	γ(SD:0.73)
Others diagnostic imaging	0.01	0.0–0.02.	γ(SD:0.08)
Complete blood cell counts	1.12	1.05–1.17	γ(SD:0.28)
Other laboratory tests	4.4	4.23–4.47	γ(SD:0.37)
Oxygen	1.37	1.28–1.45	γ(SD:0.41)
Nebulization	16.23	1.28–1.45	γ(SD:4.52)
LEV	1.1	1.07–1.13	γ(SD:0.16)
Antibiotics systemics	1.21	1.11–1.30	γ(SD:0.49)
Systemic o Inhaled Corticosteroids	0.08	0.0–0.90	γ(SD:4.18)
Bronchodilators	0.04	0.03–0.04	γ(SD:0.02)
Other drugs	0.65	0.60–0.68	γ(SD:0.04)
Medical devices	10.24	9.71–10.76	γ(SD:2.66)
Indirect cost patient-day	17.24	16.38–18.07	γ(SD:4.30)

### Sensitivity analyses

To explore the model inputs’ parameter uncertainty, a probabilistic sensitivity analysis was conducted by randomly sampling from each of the parameter distributions (beta distribution in the case of relative risk and utilities, Dirichlet distribution for multinomial data in the case of transition probabilities, and gamma distribution in the case of costs). The expected costs and expected QALYs for each treatment strategy were calculated using that combination of parameter values in the model. This process was replicated one thousand times (i.e., second-order Monte Carlo simulation) for each treatment option, resulting in the expected cost-utility. Decision uncertainty is represented in the cost-effectiveness acceptability frontiers, which plot the probability that the treatment strategy with the maximum expected net monetary benefit is the most cost-effective over a range of willingness-to-pay threshold values. Net monetary benefit was calculated by multiplying effect by societal willingness to pay and subtracting cost, with willingness-to-pay set at a ratio of US$ 20,000 per QALY. This value is the willingness-to-pay equivalent to three times the Colombian per capita gross domestic product [21]. We estimated the expected value of perfect information (EVPI). The EVPI is the maximum value that the health care system would be willing to pay for additional evidence to inform the reimbursement decision in the future. The population expected value of perfect information (PEVPI) was calculated to inform the expected cost of uncertainty (expected opportunity loss surrounding the decision) [23]. Microsoft Exel® was used in all analyses.

## Results

The model showed that FeNO was associated with lower total costs than standard therapy (US $1333 vs. US $1452 average cost per patient) and higher QALYs (0.93 vs 0.92 average per patient), showing its dominance. A position of dominance negates the need to calculate an incremental cost-effectiveness ratio (Table 3).

**Table 3:** Cost-effectiveness of FeNO-SC vs SC group

Strategy	Cost	Difference	QUALYs	Difference	C/E	Marg C/E
FeNO used in asthma management	1.33357		0.9395		1419.4	
Standard asthma management	1.45238	− 118.81	0.9233	0.016	1573	Dominated

### Sensitivity analyses

**Fig. 3:**
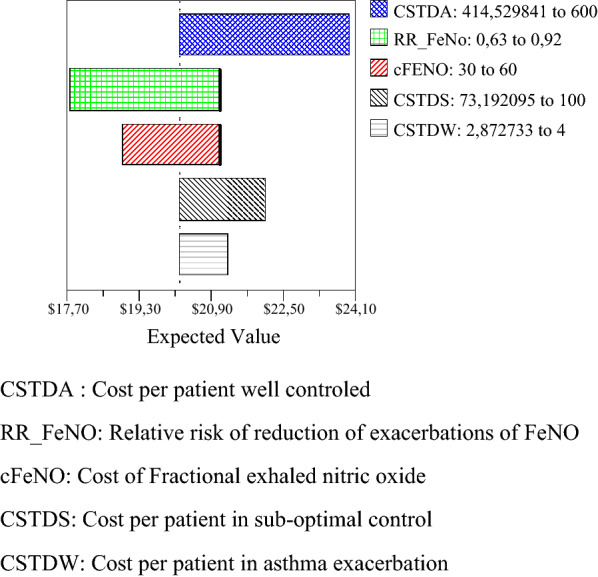
Tornado diagram

**Fig. 4:**
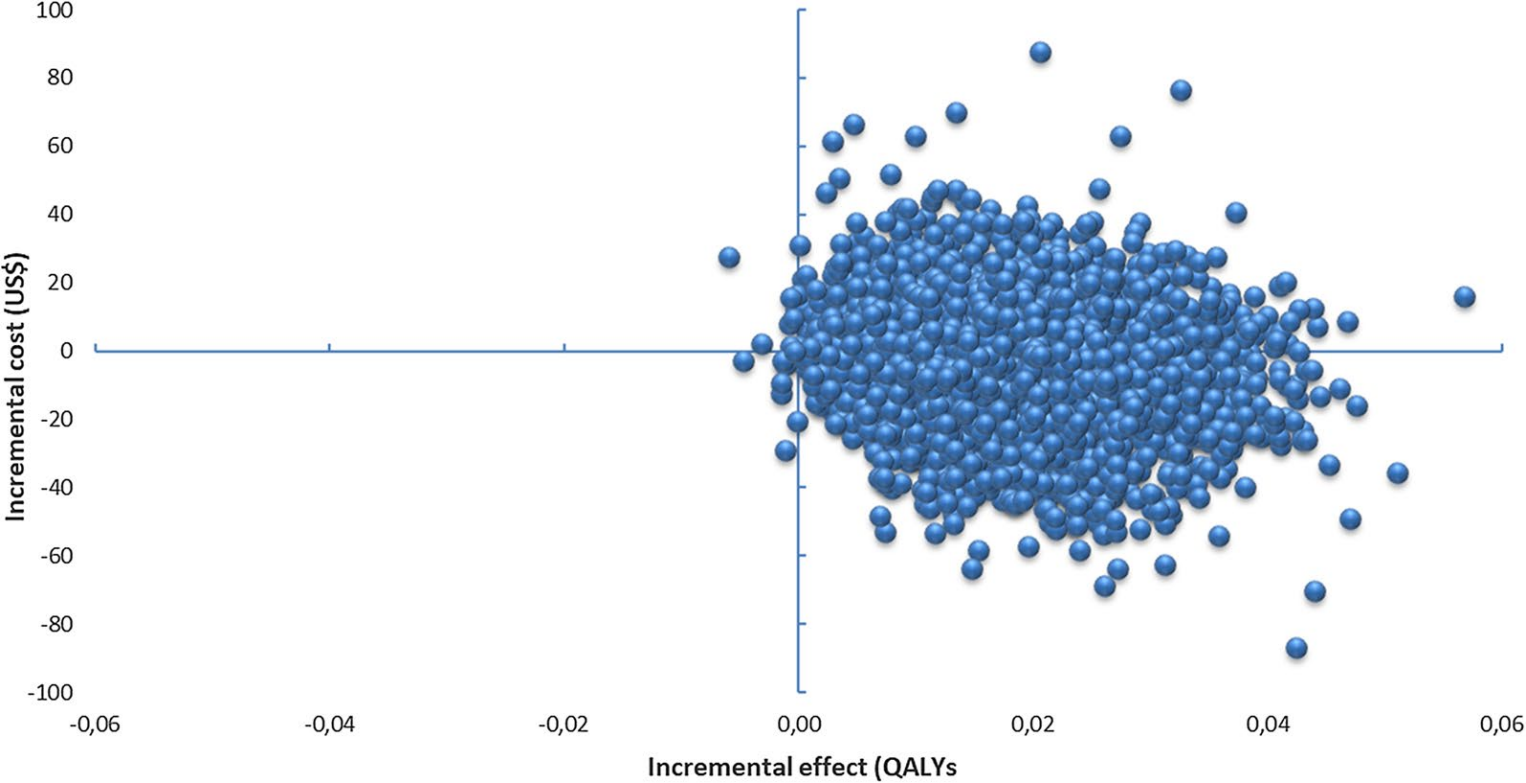
Cost effectiveness plane

**Fig. 5:**
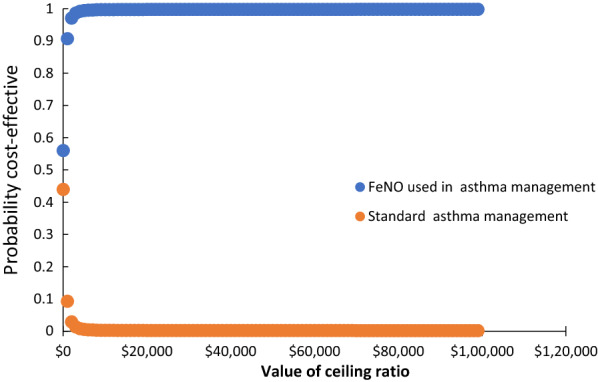
Acceptability curve

One-way sensitivity analyses showed that the probability of hospitalization has the highest impact on the outcome, but FENO was the dominant strategy in all probability ranges analyzed, Fig. 3. The results of the probabilistic sensitivity analysis are graphically represented in the cost-effectiveness plane (Fig. 4**)**. 53.82% of simulations were graphed in quadrant 2 (lower cost, high QALYs) and 45.97% were graphed in quadrant 1 of this plane (high cost, high QALYs). The 95% CI for the cost per patient treated with FeNO compared to those treated with standard therapy was US$ 1331 to 1335 and US$ 1449 to 1454, respectively. The 95% CI for QALYs per patient was 0.93 to 0.94 and 0.91 to 0.92, respectively. The net monetary benefits of FeNO were higher than those for standard therapy (US$ 16,591 vs US$ 16,885). The expected net benefit with perfect information was US$ 16,885.9 and the expected value of perfect information was US$ 0.074. The cost-effectiveness acceptability curve shows that the probability that FeNO provides a more cost-effective use of resources compared with standard therapy exceeds 99% for all willingness-to-pay thresholds (Fig. 5**)**. The population expected value of perfect information (EVPIP) for the threshold of US$ 20 was US$ 1,030,015.

## Discussion

Our study suggests that FeNO achieves better outcomes at a lower cost over standard treatment in patients under 18 with mild to moderate allergic asthma. These better outcomes are due to reductions in the likelihood of asthma exacerbations and suboptimal asthma control, which has led to a consequent increase of patients with well-controlled asthma. The magnitude of annual cost savings for the health system (US$ 118 per patient) is not negligible if we consider that this disease affects between 10 to 13% of children, with only 2.4% of them meeting the criteria for total asthma control in Colombia.

Our findings are in-line with previous studies. Beerthuizen et al. assessed the cost-effectiveness of web-based monthly monitoring and of 4-monthly monitoring of FeNO compared to standard care [24]. This economic evaluation was performed alongside a multi-center RCT with a 1-year follow-up and included 272 children aged between 4 and 18 years of age. The FeNO-based strategy had 83% chance of being most cost-effective at €40,000/QALY from a societal perspective [18]. Berg et al. assessed the cost-effectiveness of FeNO measurements with NIOX MINO in the diagnosis of asthma and in optimizing asthma management using the expected reimbursement price of the device [5]. In this study, the use of FeNO measurement in treatment decisions was less costly than asthma management based on standard guidelines (in mild to severe patients, asthma management with FeNO measurement instead of standard guidelines resulted in cost-savings of €30 per patient and year, in a more severe population, management with FeNO measurement would save costs of €160 per patient) and provided similar health benefits [7]. Brooks et al. examined the impact of FeNO monitoring on the cost-effectiveness of asthma management compared with management without FeNO [7]. FeNO had decreased expected annual expenditure per patient (US$ 2,228) and increased expected annual QALYs per patient (0.844) compared with the current standard care (US$ 2,637 and 0.767) [9]. Price et al. determined the cost-effectiveness of FeNO measurement using a hand-held monitor (NIOX MINO) at a reimbursement price of £23 for asthma diagnosis and management in the UK [25]. Asthma management using FeNO measurement instead of lung function testing resulted in annual cost-savings of £341 and 0.06 QALYs gained for patients with mild to severe asthma and cost-savings of £554 and 0.004 QALYs gained for those with moderate to severe asthma [19]. Sabatelli et al. evaluated the cost-effectiveness and budget impact of FeNO monitoring for management of adult asthma in Spain over a 1-year period [6]. Adding FeNO to standard asthma care saved €62.53 per patient-year and improved QALYs by 0.026 per patient-year. The budget impact analysis revealed a potential net yearly saving of €129 million if FeNO monitoring had been used in primary care settings in Spain. Similarly, Harnan et al. assessed the cost-effectiveness of the hand-held electrochemical devices NIOX MINO® (Aerocrine, Solna, Sweden), NIOX VERO® (Aerocrine) and NO breath® (Bedfont Scientific, Maidstone, UK) for the diagnosis and management of asthma [8]. The novo management model indicated that the ICER of guidelines plus FeNO monitoring using NO breath compared with guidelines alone in children is expected to be approximately £45,200 per QALY gained, concluding that FeNO-guided management has the potential to be cost-effective, although this is largely dependent on the duration of the effect.

The latest version of the Global Initiative for Asthma refers to children: “FeNO-guided treatment significantly reduces exacerbation rates compared with guidelines-based treatment (Evidence A). However, further studies are needed to identify the populations most likely to benefit from FeNO-guided treatment and to determine the optimal frequency of FeNO monitoring” [1]. References that support this statement only include RCTs, with no economic evaluations corroborating it. The dynamic between clinical research on effectiveness and the research of efficiency must be coordinated and synchronous in order to make recommendations from the individual to the public health level. The transferability of economic evaluations is clearly complex, but this situation highlights the need to assess health technologies in the clinical guidelines that do not only evaluate effectiveness or safety, but that also review economical topics to increase the level of recommendations in clinical guidelines.

A very important aspect of our model is that it was robust to changing the values of the model's utilities, probabilities, and costs using the Markov model’s one-way and probabilistic sensitivity analysis. FeNO was always the cost-effectiveness strategy in all value ranges of utilities, probabilities, and costs. Moreover, FeNO was always the cost-effectiveness strategy in all ranges of thresholds evaluated with a low population EVPI. These findings in the sensitivity analysis are of cardinal importance in our study because many of the inputs were extracted from literature, which was all hospital-based and undertaken in affluent countries. They also allowed decision-making with an estimated degree of uncertainty in each cost parameter or QALYs per strategy.

Our study has some limitations. The cost data were collected retrospectively. Asthma treatment and the costs in question, including hospital prices, did not markedly change to date. Furthermore, our country has been characterized by having very low price variation in the last 10 years, especially in terms of health services [20]. In addition, we use utilities extracted from the literature and not estimated directly from our population. As was mentioned previously, the reliability and robustness of the results were evaluated by sensitivity analyses.

## Conclusion

Asthma management using fractional exhaled nitric oxide monitoring was cost-effective for treating patients aged between 4 and 18 with mild to moderate allergic asthma. Our study suggests evidence that could be used by decision-makers to improve clinical practice guidelines, although it should be replicated in different clinical settings.

## Data Availability

The raw data supporting your findings can be request to http://ciemto.medicinaudea.co/.

## Abbreviations

FeNO: Fractional exhaled nitric oxide
FeNO-SC: SC with FeNO monitoring to guide asthma management in Children
QALYs: Quality-adjusted life years
RCT: Randomized clinical trials
